# OLDER WORKERS’ AND EMPLOYERS’ PERSPECTIVE ON EXTENDING WORKING LIFE: THE CASE OF POLAND

**DOI:** 10.1093/geroni/igad104.1790

**Published:** 2023-12-21

**Authors:** Jolanta Perek-Białas, Anna Urbaniak, Natalia Krygowska-Nowak, Maria Varlamova

**Affiliations:** Jagiellonian University, Kraków, Malopolskie, Poland; Jagiellonian University Krakow, Krakow, Malopolskie, Poland; Jagiellonian University Krakow, Krakow, Malopolskie, Poland; Jagiellonian University Krakow, Krakow, Malopolskie, Poland

## Abstract

Extending working life is considered an adequate policy response to population aging and workforce shrinkage. However, in some countries, like Poland, there are still no clear mechanisms supporting extending working life. Poland makes an interesting case, as while the eligible retirement age is one of the lowest in Europe, no plans to increase it are set. The aim of this paper is to present perspectives of older workers and employers on extending working life. We identify factors that constitute supportive and unsupportive working environments from the perspective of older Polish workers and show how employers perceive workers over 50 in the workplace. First, the qualitative component of the programme EIWO ‘Exclusion and Inequality in Late Working Life’ is analysed. We draw from 25 semi-structured interviews conducted between June and November of 2021 with men and women aged 55-75 years old working in private and public sectors in Poland. Secondly, the probability survey among Polish employers (n=1,037) was analysed to show the attitudes and perceptions of employers. Older workers working in the public sector feel less satisfied with the financial aspects of employment, less motivated to extend their working lives, and more often describe practices of pushing older workers out of the labour market. The analyses of employees give matching results. These findings highlight that in the absence of a governmental strategy, extending working life is more dependent on employers and employees that might differ among sectors of employment.

